# Bringing cognitive behavioral therapy for nightmares to patients with trauma: increasing access and feasibility via primary and acute care

**DOI:** 10.1007/s44470-026-00108-5

**Published:** 2026-07-07

**Authors:** Anthony N. Reffi, Jennifer Peltzer-Jones, David A. Moore, Jeffrey L. Johnson, Christopher L. Drake

**Affiliations:** 1 Sleep Disorders & Research Center, Henry Ford Health, Detroit, MI USA; 2 Department of Psychiatry, Michigan State University College of Human Medicine, Grand Rapids, MI USA; 3 Division of Acute Care Surgery, Department of Surgery, Henry Ford Hospital, Detroit, MI USA; 4 Department of Emergency Medicine, Henry Ford Health, Detroit, MI USA; 5 Department of Psychiatry and Behavioral Neurosciences, Wayne State University, Detroit, MI USA; 6 Division of Consultation Liaison Psychiatry, Department of Psychiatry and Behavioral Health, Henry Ford Hospital, Detroit, MI USA

Nightmares affect one in six US veterans and are associated with increased risk of posttraumatic stress disorder (PTSD) and suicidal thoughts and behaviors [1]. Although traditionally viewed as secondary symptoms of PTSD, nightmares often do not respond to first-line PTSD treatments and are associated with worse treatment outcomes [2–4], highlighting a need to target them directly. However, nightmares are seldom reported to providers and remain largely untreated [5, 6], spurring recent efforts to proactively screen for nightmares and increase access to nightmare-focused interventions.

Cognitive Behavioral Therapy for Nightmares (CBT-N) is a recently standardized protocol for nightmares that integrates the key components of extant nightmare therapies [7, 8], including exposure to nightmare content, nightmare rescripting, and imagery rehearsal [9–11]. Historically, the literature on behavioral treatment for nightmares has been mired by a lack of clarity, such as the inconsistent use of treatment components across manuals of the same name [7]. The impetus for formalizing these components under the CBT-N umbrella was, in part, to reduce this confusion, and make it easier for clinicians to deliver nightmare therapy [12]. Given the recency of the CBT-N manual, it remains to be seen how to bring this treatment to the patients who need it most, such as veterans, and how the protocol is received by providers working across different settings.

As reported in their study recently published in the *Journal of Clinical Sleep Medicine*, Bolstad et al. [13] have begun answering these questions by bringing CBT-N into the Primary Care Mental Health Integration (PCMHI) clinic at the Veterans Health Administration (VHA). The PCMHI setting is notable for at least two reasons. First, behavioral health providers in this setting are embedded in primary care clinics, which do not typically assess nightmares. This integration allows behavioral health providers to see a much larger scope of patients than providers in traditional mental health clinics, including patients who may be reluctant to seek psychotherapy. Thus, implementing CBT-N in PCMHI has potential to expand access to veterans at scale.

Second, PCMHI follows a brief model of care, with patients receiving an average of 2–3 behavioral health sessions lasting 20–30 min each. This format is a stark departure from traditional mental health clinics which typically entail at least six 50- to 60-min sessions. Indeed, CBT-N involves six 60- to 90-min sessions [7]. Therefore, evaluating the implementation of CBT-N in PCMHI is ideal for revealing how the treatment should be adapted for this fast-paced setting.

The researchers found PCMHI clinicians perceived greater benefits of CBT-N for veterans compared to clinicians in other VHA mental health settings (e.g., PTSD Clinical Team) [13]. PCMHI clinicians detected greater change in how the veterans related to their nightmares, such as feeling less afraid of them. PCMHI clinicians also perceived greater change in veterans’ mental health more broadly relative to clinicians in other settings, such as improvements in trauma-related and/or depressive symptoms and quality of life. Despite these perceived benefits, *PCMHI clinicians rated CBT-N to be less appropriate and feasible to deliver compared to clinicians in other settings*, with 57% of PCMHI clinicians citing time constraints [13]. In turn, Bolstad et al. [13] propose adapting CBT-N for PCMHI by having shorter, more frequent sessions.

It is possible that veterans in PCMHI were perceived to do better because they were less distressed than those seen in more traditional mental health settings. Perhaps PCMHI clinicians intervened early in the nightmare-disease course, reducing the risk of worsening mental health, whereas clinicians in other settings likely saw patients who had already reached clinically significant distress. This interpretation assumes nightmares are sentinel symptoms, the treatment of which produces downstream preventive effects, which could not be known from this study.

Our team is actively testing this proposition in patients exposed to recent trauma. Nightmares affect anywhere from 46% to 73% of acute trauma patients [14, 15] and serve as a prodrome for PTSD [14–17] and suicidal ideation [18]. We are piloting a randomized controlled trial of behavioral sleep interventions within the immediate aftermath of trauma to halt the progression to post-trauma pathology (NCT07121270) [19].

We enroll patients discharged from our 13 Emergency Departments (ED) across our health system within 72 h of trauma. Patients are randomized and triaged based on symptoms to CBT-I or CBT-N, or a sleep education control with or without nightmare education. Thus far, we have randomized 34 patients (mostly physical assault, 47.1%) and have found a significant demand for nightmare treatment, *with 85.1% of acute trauma patients expressing interest in nightmare therapy*. We deliver our interventions via telehealth video to reduce barriers to care and have seen an encouraging 76.5% of those randomized initiate treatment, whereas roughly half of patients discharged from the ED attend an outpatient mental health visit [20–22].

Moreover, 100% of patients found it “very easy” to attend our video sessions post-trauma, and 71.4% said it would have been difficult to attend sessions had they been in-person. When asked about preferred format for future services, most patients preferred video (85.7%) over in-person sessions (14.3%), and 100% preferred video sessions over a mobile app (0.0%). Future analyses include evaluating the preventive effects of CBT-I or CBT-N delivered early after trauma on future PTSD relative to control.

Ultimately, our work dovetails with the valuable findings of Bolstad et al. [13], who took an important step toward increasing access to nightmare therapy for veterans through the primary care setting. Providers working across different settings are encouraged to bring CBT-N into their practice as well, including sleep medicine clinics [23], and can offer treatment to patients experiencing *D*reams that cause *A*wakenings, are at least partially *R*emembered, and cause *C*linically significant impairment (“DARC”) [7].

Remaining questions include if and how CBT-N should be adapted for active-duty military [24], first responders [25], and inpatients hospitalized following traumatic injury who are unable to write about nightmares due to physical injuries. Taken together, these studies remind us that not all who need mental health care are in the waiting rooms of our behavioral health clinics. As providers and researchers, we ought to be thoughtful about meeting patients where they are, and adapting our treatments to fit those contexts, wherever they may be.

## Data Availability

The data that support the findings of this study are available from the corresponding author upon reasonable request.
